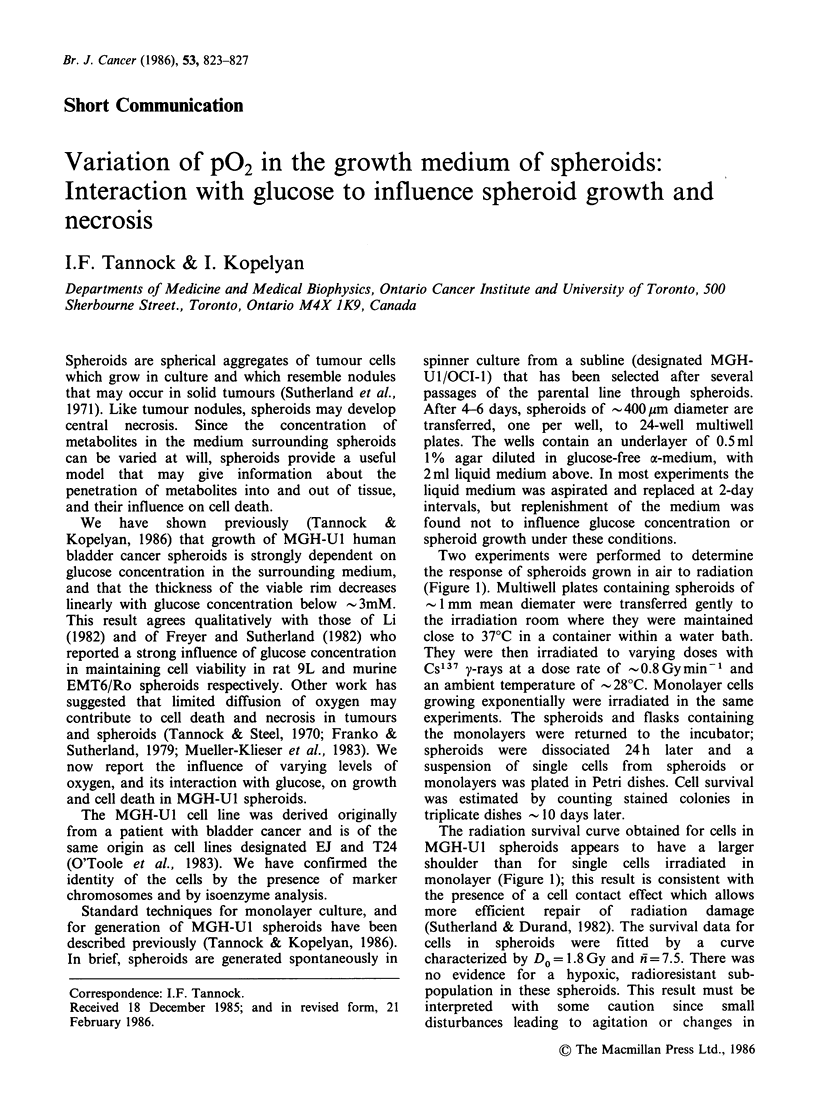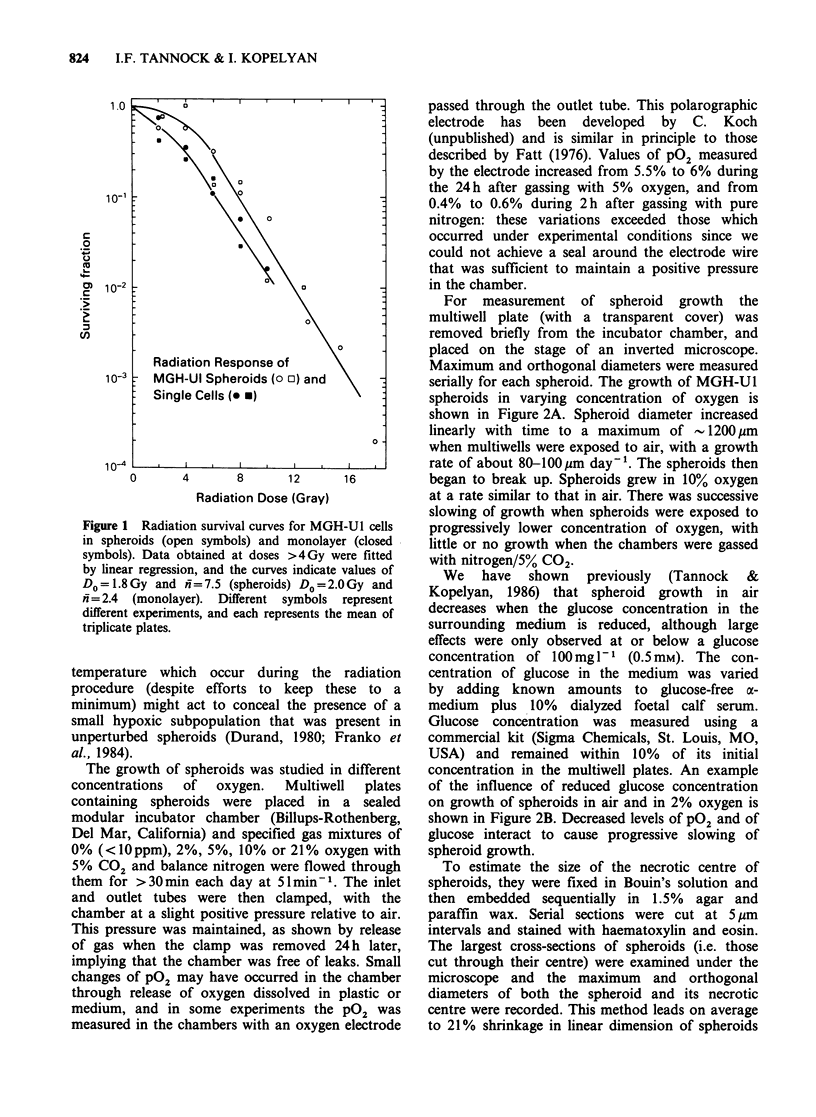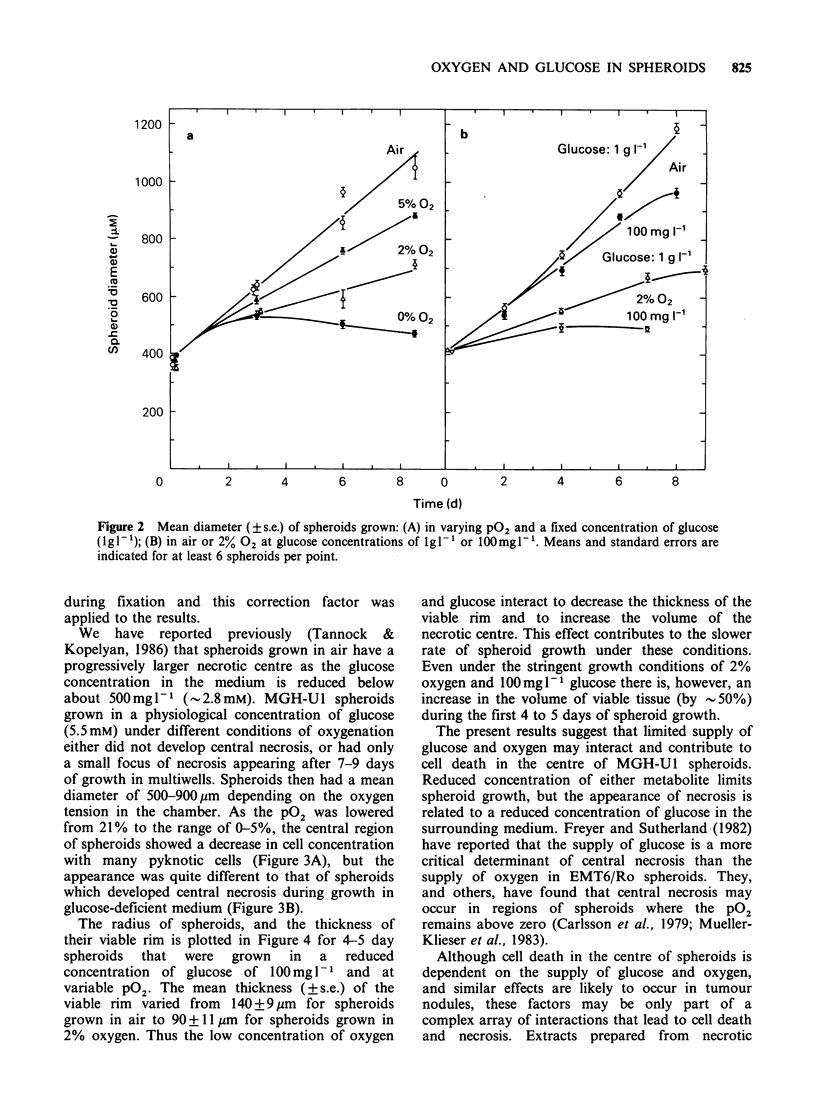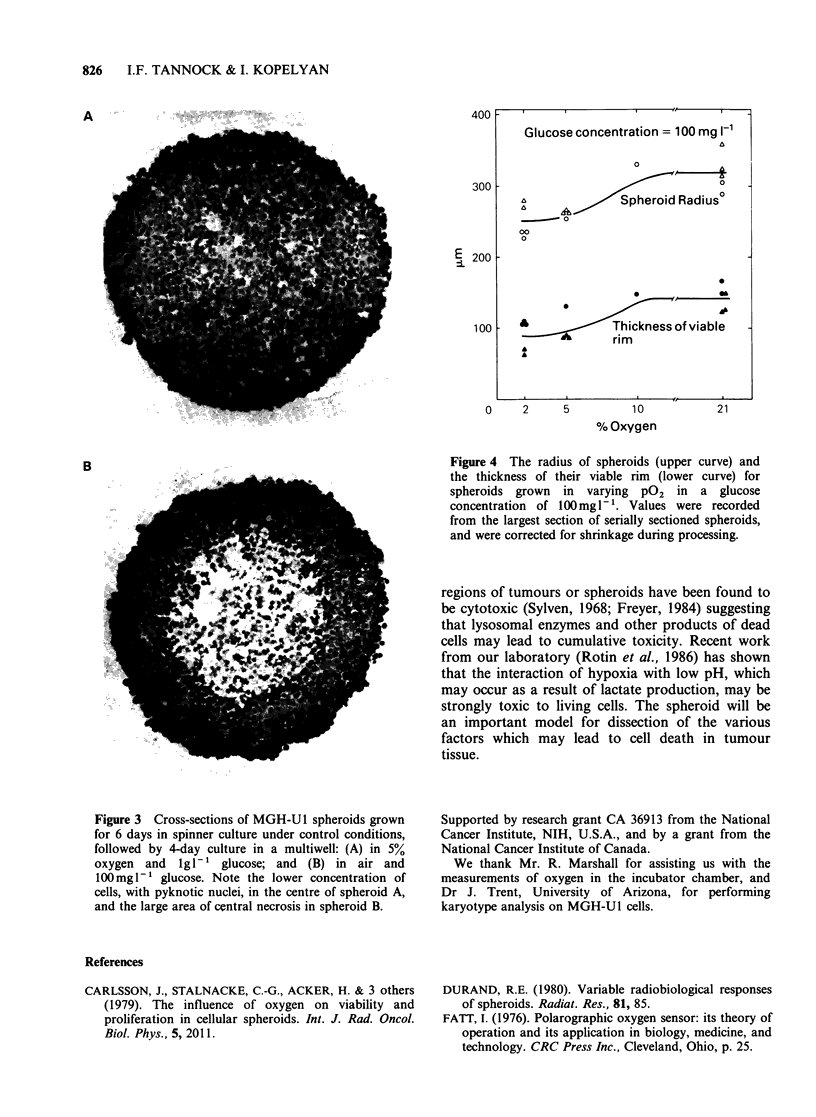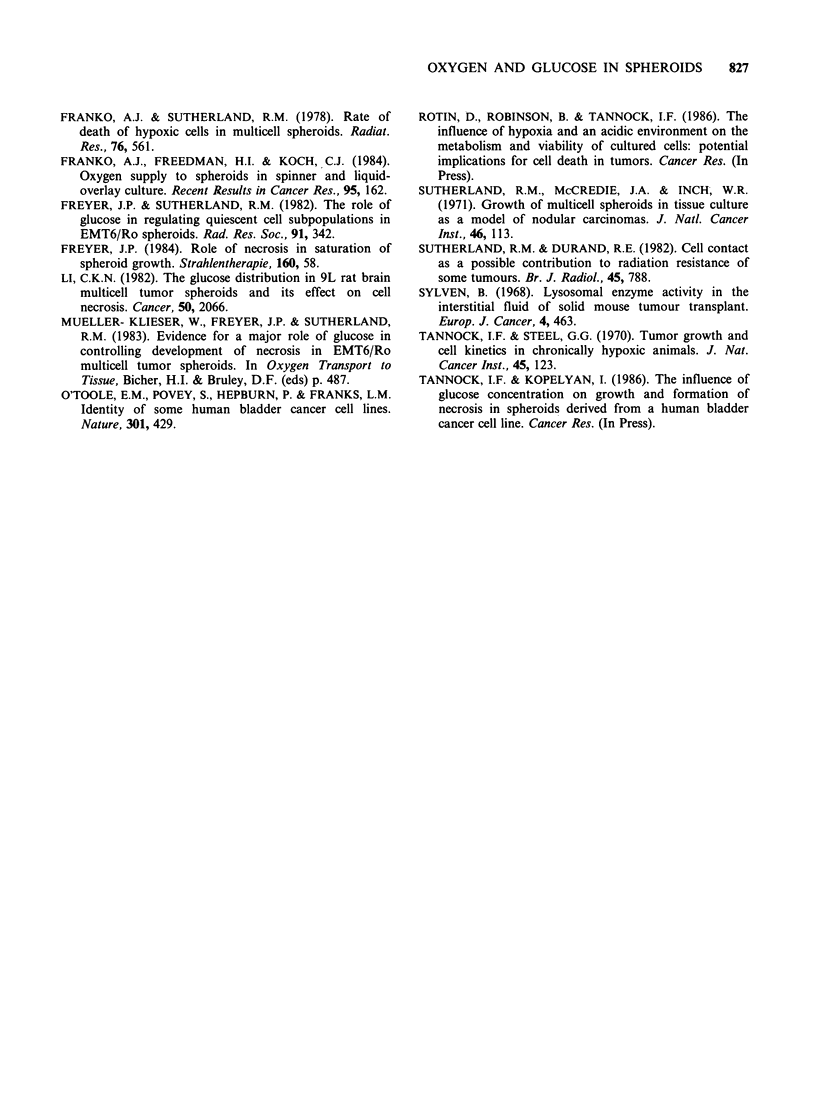# Variation of pO2 in the growth medium of spheroids: interaction with glucose to influence spheroid growth and necrosis.

**DOI:** 10.1038/bjc.1986.139

**Published:** 1986-06

**Authors:** I. F. Tannock, I. Kopelyan

## Abstract

**Images:**


*Br. J. Cancer* (1986), **53**, 823–827

### Short Communication

### Variation of $pO_2$ in the growth medium of spheroids: Interaction with glucose to influence spheroid growth and necrosis

I.F. Tannock & I. Kopelyan

*Departments of Medicine and Medical Biophysics, Ontario Cancer Institute and University of Toronto, 500 Sherbourne Street., Toronto, Ontario M4X 1K9, Canada*

Spheroids are spherical aggregates of tumour cells which grow in culture and which resemble nodules that may occur in solid tumours (Sutherland *et al.*, 1971). Like tumour nodules, spheroids may develop central necrosis. Since the concentration of metabolites in the medium surrounding spheroids can be varied at will, spheroids provide a useful model that may give information about the penetration of metabolites into and out of tissue, and their influence on cell death.

We have shown previously (Tannock & Kopelyan, 1986) that growth of MGH-U1 human bladder cancer spheroids is strongly dependent on glucose concentration in the surrounding medium, and that the thickness of the viable rim decreases linearly with glucose concentration below ~3mM. This result agrees qualitatively with those of Li (1982) and of Freyer and Sutherland (1982) who reported a strong influence of glucose concentration in maintaining cell viability in rat 9L and murine EMT6/Ro spheroids respectively. Other work has suggested that limited diffusion of oxygen may contribute to cell death and necrosis in tumours and spheroids (Tannock & Steel, 1970; Franko & Sutherland, 1979; Mueller-Klieser *et al.*, 1983). We now report the influence of varying levels of oxygen, and its interaction with glucose, on growth and cell death in MGH-U1 spheroids.

The MGH-U1 cell line was derived originally from a patient with bladder cancer and is of the same origin as cell lines designated EJ and T24 (O'Toole *et al.*, 1983). We have confirmed the identity of the cells by the presence of marker chromosomes and by isoenzyme analysis.

Standard techniques for monolayer culture, and for generation of MGH-U1 spheroids have been described previously (Tannock & Kopelyan, 1986). In brief, spheroids are generated spontaneously in spinner culture from a subline (designated MGH-U1/OCI-1) that has been selected after several passages of the parental line through spheroids. After 4–6 days, spheroids of ~400 $\mu$m diameter are transferred, one per well, to 24-well multiwell plates. The wells contain an underlayer of 0.5 ml 1% agar diluted in glucose-free $\alpha$-medium, with 2 ml liquid medium above. In most experiments the liquid medium was aspirated and replaced at 2-day intervals, but replenishment of the medium was found not to influence glucose concentration or spheroid growth under these conditions.

Two experiments were performed to determine the response of spheroids grown in air to radiation (Figure 1). Multiwell plates containing spheroids of ~1 mm mean diemater were transferred gently to the irradiation room where they were maintained close to 37°C in a container within a water bath. They were then irradiated to varying doses with $Cs^{137}$ $\gamma$-rays at a dose rate of ~0.8 Gy min$^{-1}$ and an ambient temperature of ~28°C. Monolayer cells growing exponentially were irradiated in the same experiments. The spheroids and flasks containing the monolayers were returned to the incubator; spheroids were dissociated 24 h later and a suspension of single cells from spheroids or monolayers was plated in Petri dishes. Cell survival was estimated by counting stained colonies in triplicate dishes ~10 days later.

The radiation survival curve obtained for cells in MGH-U1 spheroids appears to have a larger shoulder than for single cells irradiated in monolayer (Figure 1); this result is consistent with the presence of a cell contact effect which allows more efficient repair of radiation damage (Sutherland & Durand, 1982). The survival data for cells in spheroids were fitted by a curve characterized by $D_0 = 1.8$ Gy and $\bar{n} = 7.5$. There was no evidence for a hypoxic, radioresistant sub-population in these spheroids. This result must be interpreted with some caution since small disturbances leading to agitation or changes in

Correspondence: I.F. Tannock.
Received 18 December 1985; and in revised form, 21 February 1986.

© The Macmillan Press Ltd., 1986

**Figure 1** Radiation survival curves for MGH-U1 cells in spheroids (open symbols) and monolayer (closed symbols). Data obtained at doses >4 Gy were fitted by linear regression, and the curves indicate values of $D_0 = 1.8$ Gy and $\bar{n} = 7.5$ (spheroids) $D_0 = 2.0$ Gy and $\bar{n} = 2.4$ (monolayer). Different symbols represent different experiments, and each represents the mean of triplicate plates.

temperature which occur during the radiation procedure (despite efforts to keep these to a minimum) might act to conceal the presence of a small hypoxic subpopulation that was present in unperturbed spheroids (Durand, 1980; Franko *et al.*, 1984).

The growth of spheroids was studied in different concentrations of oxygen. Multiwell plates containing spheroids were placed in a sealed modular incubator chamber (Billups-Rothenberg, Del Mar, California) and specified gas mixtures of 0% (<10 ppm), 2%, 5%, 10% or 21% oxygen with 5% $CO_2$ and balance nitrogen were flowed through them for >30 min each day at $5\,l\,min^{-1}$. The inlet and outlet tubes were then clamped, with the chamber at a slight positive pressure relative to air. This pressure was maintained, as shown by release of gas when the clamp was removed 24 h later, implying that the chamber was free of leaks. Small changes of $pO_2$ may have occurred in the chamber through release of oxygen dissolved in plastic or medium, and in some experiments the $pO_2$ was measured in the chambers with an oxygen electrode passed through the outlet tube. This polarographic electrode has been developed by C. Koch (unpublished) and is similar in principle to those described by Fatt (1976). Values of $pO_2$ measured by the electrode increased from 5.5% to 6% during the 24 h after gassing with 5% oxygen, and from 0.4% to 0.6% during 2 h after gassing with pure nitrogen: these variations exceeded those which occurred under experimental conditions since we could not achieve a seal around the electrode wire that was sufficient to maintain a positive pressure in the chamber.

For measurement of spheroid growth the multiwell plate (with a transparent cover) was removed briefly from the incubator chamber, and placed on the stage of an inverted microscope. Maximum and orthogonal diameters were measured serially for each spheroid. The growth of MGH-U1 spheroids in varying concentration of oxygen is shown in Figure 2A. Spheroid diameter increased linearly with time to a maximum of $\sim 1200\,\mu m$ when multiwells were exposed to air, with a growth rate of about 80–100 $\mu m$ $day^{-1}$. The spheroids then began to break up. Spheroids grew in 10% oxygen at a rate similar to that in air. There was successive slowing of growth when spheroids were exposed to progressively lower concentration of oxygen, with little or no growth when the chambers were gassed with nitrogen/5% $CO_2$.

We have shown previously (Tannock & Kopelyan, 1986) that spheroid growth in air decreases when the glucose concentration in the surrounding medium is reduced, although large effects were only observed at or below a glucose concentration of $100\,mg\,l^{-1}$ (0.5 mM). The concentration of glucose in the medium was varied by adding known amounts to glucose-free $\alpha$-medium plus 10% dialyzed foetal calf serum. Glucose concentration was measured using a commercial kit (Sigma Chemicals, St. Louis, MO, USA) and remained within 10% of its initial concentration in the multiwell plates. An example of the influence of reduced glucose concentration on growth of spheroids in air and in 2% oxygen is shown in Figure 2B. Decreased levels of $pO_2$ and of glucose interact to cause progressive slowing of spheroid growth.

To estimate the size of the necrotic centre of spheroids, they were fixed in Bouin's solution and then embedded sequentially in 1.5% agar and paraffin wax. Serial sections were cut at 5 $\mu m$ intervals and stained with haematoxylin and eosin. The largest cross-sections of spheroids (i.e. those cut through their centre) were examined under the microscope and the maximum and orthogonal diameters of both the spheroid and its necrotic centre were recorded. This method leads on average to 21% shrinkage in linear dimension of spheroids

**Figure 2** Mean diameter ($\pm$s.e.) of spheroids grown: (A) in varying $pO_2$ and a fixed concentration of glucose ($1 g l^{-1}$); (B) in air or 2% $O_2$ at glucose concentrations of $1 g l^{-1}$ or $100 mg l^{-1}$. Means and standard errors are indicated for at least 6 spheroids per point.

during fixation and this correction factor was applied to the results.

We have reported previously (Tannock & Kopelyan, 1986) that spheroids grown in air have a progressively larger necrotic centre as the glucose concentration in the medium is reduced below about $500 mg l^{-1}$ ($\sim$2.8 mM). MGH-U1 spheroids grown in a physiological concentration of glucose (5.5 mM) under different conditions of oxygenation either did not develop central necrosis, or had only a small focus of necrosis appearing after 7–9 days of growth in multiwells. Spheroids then had a mean diameter of 500–900 μm depending on the oxygen tension in the chamber. As the $pO_2$ was lowered from 21% to the range of 0–5%, the central region of spheroids showed a decrease in cell concentration with many pyknotic cells (Figure 3A), but the appearance was quite different to that of spheroids which developed central necrosis during growth in glucose-deficient medium (Figure 3B).

The radius of spheroids, and the thickness of their viable rim is plotted in Figure 4 for 4–5 day spheroids that were grown in a reduced concentration of glucose of $100 mg l^{-1}$ and at variable $pO_2$. The mean thickness ($\pm$s.e.) of the viable rim varied from $140 \pm 9$ μm for spheroids grown in air to $90 \pm 11$ μm for spheroids grown in 2% oxygen. Thus the low concentration of oxygen and glucose interact to decrease the thickness of the viable rim and to increase the volume of the necrotic centre. This effect contributes to the slower rate of spheroid growth under these conditions. Even under the stringent growth conditions of 2% oxygen and $100 mg l^{-1}$ glucose there is, however, an increase in the volume of viable tissue (by $\sim$50%) during the first 4 to 5 days of spheroid growth.

The present results suggest that limited supply of glucose and oxygen may interact and contribute to cell death in the centre of MGH-U1 spheroids. Reduced concentration of either metabolite limits spheroid growth, but the appearance of necrosis is related to a reduced concentration of glucose in the surrounding medium. Freyer and Sutherland (1982) have reported that the supply of glucose is a more critical determinant of central necrosis than the supply of oxygen in EMT6/Ro spheroids. They, and others, have found that central necrosis may occur in regions of spheroids where the $pO_2$ remains above zero (Carlsson *et al.*, 1979; Mueller-Klieser *et al.*, 1983).

Although cell death in the centre of spheroids is dependent on the supply of glucose and oxygen, and similar effects are likely to occur in tumour nodules, these factors may be only part of a complex array of interactions that lead to cell death and necrosis. Extracts prepared from necrotic

**Figure 4** The radius of spheroids (upper curve) and the thickness of their viable rim (lower curve) for spheroids grown in varying $pO_2$ in a glucose concentration of $100 \, mg \, l^{-1}$. Values were recorded from the largest section of serially sectioned spheroids, and were corrected for shrinkage during processing.

**Figure 3** Cross-sections of MGH-U1 spheroids grown for 6 days in spinner culture under control conditions, followed by 4-day culture in a multiwell: (A) in 5% oxygen and $1 \, g \, l^{-1}$ glucose; and (B) in air and $100 \, mg \, l^{-1}$ glucose. Note the lower concentration of cells, with pyknotic nuclei, in the centre of spheroid A, and the large area of central necrosis in spheroid B.

regions of tumours or spheroids have been found to be cytotoxic (Sylven, 1968; Freyer, 1984) suggesting that lysosomal enzymes and other products of dead cells may lead to cumulative toxicity. Recent work from our laboratory (Rotin *et al.*, 1986) has shown that the interaction of hypoxia with low pH, which may occur as a result of lactate production, may be strongly toxic to living cells. The spheroid will be an important model for dissection of the various factors which may lead to cell death in tumour tissue.

Supported by research grant CA 36913 from the National Cancer Institute, NIH, U.S.A., and by a grant from the National Cancer Institute of Canada.

We thank Mr. R. Marshall for assisting us with the measurements of oxygen in the incubator chamber, and Dr J. Trent, University of Arizona, for performing karyotype analysis on MGH-U1 cells.

### References

CARLSSON, J., STALNACKE, C.-G., ACKER, H. & 3 others (1979). The influence of oxygen on viability and proliferation in cellular spheroids. *Int. J. Rad. Oncol. Biol. Phys.*, **5**, 2011.

DURAND, R.E. (1980). Variable radiobiological responses of spheroids. *Radiat. Res.*, **81**, 85.

FATT, I. (1976). Polarographic oxygen sensor: its theory of operation and its application in biology, medicine, and technology. *CRC Press Inc.*, Cleveland, Ohio, p. 25.

FRANKO, A.J. & SUTHERLAND, R.M. (1978). Rate of death of hypoxic cells in multicell spheroids. *Radiat. Res.*, **76**, 561.

FRANKO, A.J., FREEDMAN, H.I. & KOCH, C.J. (1984). Oxygen supply to spheroids in spinner and liquid-overlay culture. *Recent Results in Cancer Res.*, **95**, 162.

FREYER, J.P. & SUTHERLAND, R.M. (1982). The role of glucose in regulating quiescent cell subpopulations in EMT6/Ro spheroids. *Rad. Res. Soc.*, **91**, 342.

FREYER, J.P. (1984). Role of necrosis in saturation of spheroid growth. *Strahlentherapie*, **160**, 58.

LI, C.K.N. (1982). The glucose distribution in 9L rat brain multicell tumor spheroids and its effect on cell necrosis. *Cancer*, **50**, 2066.

MUELLER- KLIESER, W., FREYER, J.P. & SUTHERLAND, R.M. (1983). Evidence for a major role of glucose in controlling development of necrosis in EMT6/Ro multicell tumor spheroids. In *Oxygen Transport to Tissue*, Bicher, H.I. & Bruley, D.F. (eds) p. 487.

O'TOOLE, E.M., POVEY, S., HEPBURN, P. & FRANKS, L.M. Identity of some human bladder cancer cell lines. *Nature*, **301**, 429.

ROTIN, D., ROBINSON, B. & TANNOCK, I.F. (1986). The influence of hypoxia and an acidic environment on the metabolism and viability of cultured cells: potential implications for cell death in tumors. *Cancer Res.* (In Press).

SUTHERLAND, R.M., McCREDIE, J.A. & INCH, W.R. (1971). Growth of multicell spheroids in tissue culture as a model of nodular carcinomas. *J. Natl. Cancer Inst.*, **46**, 113.

SUTHERLAND, R.M. & DURAND, R.E. (1982). Cell contact as a possible contribution to radiation resistance of some tumours. *Br. J. Radiol.*, **45**, 788.

SYLVEN, B. (1968). Lysosomal enzyme activity in the interstitial fluid of solid mouse tumour transplant. *Europ. J. Cancer*, **4**, 463.

TANNOCK, I.F. & STEEL, G.G. (1970). Tumor growth and cell kinetics in chronically hypoxic animals. *J. Nat. Cancer Inst.*, **45**, 123.

TANNOCK, I.F. & KOPELYAN, I. (1986). The influence of glucose concentration on growth and formation of necrosis in spheroids derived from a human bladder cancer cell line. *Cancer Res.* (In Press).